# FAMILY CAREGIVING DURING THE COVID-19 PANDEMIC FOR OLDER ADULTS WITH DEMENTIA

**DOI:** 10.1093/geroni/igad104.0343

**Published:** 2023-12-21

**Authors:** Emily Wiemers, I-Fen Lin

## Abstract

Family caregiving plays a crucial role in meeting the care needs of older adults, especially those living with Alzheimer’s disease and related dementias (ADRD). The COVID-19 disrupted an already fragile care landscape by reducing the desirability of nursing home care and the availability of paid caregivers in the home and by destabilizing family caregiving arrangements. Older adult living with ADRD may be at higher risk for destabilized care and more vulnerable to adverse effects from the COVID-19 pandemic’s disruption to the long-term care landscape. This symposium draws on five papers providing qualitative and quantitative evidence of how care arrangements shifted during the COVID-19 pandemic among older adults with ADRD. Patterson and colleagues set the stage for understanding the impacts of the pandemic on family caregiving for older adults with dementia by providing estimates of how many families and households include someone with dementia. Choi and colleagues use a mixed-methods approach to show that family members provided more care during than before the pandemic, especially coresident family members. Lin and colleagues use quantitative evidence to examine how pandemic-specific needs and help from nonresident adult children varied by older adults’ cognitive functioning. Leggett and Tsuker examine the consequences of the pandemic for caregiver stress across different ADRD caregiver relationship types. Finally, Wiemers and colleagues extend the focus on unpaid family caregivers to include paid care and show how tradeoffs between paid and unpaid care for older adults with ADRD were affected by pandemic severity.

